# Supplementary material for: A chemical proteomic atlas of brain serine hydrolases identifies cell type-specific pathways regulating neuroinflammation
Source: eLife. 2016 Jan 18;5:e12345. doi: 10.7554/eLife.12345 (PMC4737654; doi:10.7554/eLife.12345)
Supplement: Figure 1—source data 1. — Peptide spectral counts (SC) of serine hydrolases in neuron, astrocyte and microglia proteomes. Average SC values of four individual ABPP-MudPIT experiments ± SEM are reported for proteins identified with a minimum of five SCs in at least one cell type. DOI: http://dx.doi.org/10.7554/eLife.12345.004 [file elife-12345-fig1-data1.docx]

**Figure 1—source data 1.** **Serine hydrolases identified in neuron, astrocyte, and microglia proteomes by ABPP-MudPIT.**

Peptide spectral counts (SC) of serine hydrolases in neuron, astrocyte and microglia proteomes. Average SC values of four individual ABPP-MudPIT experiments ± SEM are reported for proteins identified with a minimum of five SCs in at least one cell type.

|  | | | | | |
| --- | --- | --- | --- | --- | --- |
| **Uniprot #** | **Common name** | **Abbreviation** | **Neuron  SC ± SEM** | **Astrocyte  SC ± SEM** | **Microglia  SC ± SEM** |
| P19096 | Fatty acid synthase | FASN | 262 ± 41 | 327 ± 53 | 256 ± 57 |
| Q9QUR6 | Prolyl endopeptidase | PREP | 255 ± 22 | 227 ± 34 | 321 ± 38 |
| Q8R2Y0 | α−β hydrolase 6 | ABHD6 | 117 ± 29 | 150 ± 23 | 60 ± 18 |
| Q61206 | Platelet-activating factor acetylhydrolase IB subunit β | PAFAH1B2 | 104 ± 10 | 37 ± 12 | 55 ± 14 |
| Q9R0P3 | S-formylglutathione hydrolase | ESD | 100 ± 4 | 328 ± 64 | 207 ± 29 |
| Q8BLF1 | Neutral cholesterol ester hydrolase 1 | NCEH1 | 80 ± 10 | 244 ± 27 | 229 ± 26 |
| Q8C167 | Prolyl endopeptidase-like | PREPL | 80 ± 12 | 96 ± 15 | 44 ± 11 |
| Q61205 | Platelet-activating factor acetylhydrolase IB subunit γ | PAFAH1B3 | 79 ± 14 | 17 ± 6 | 57 ± 16 |
| Q9WTL7 | Acyl-protein thioesterase 2 | LYPLA2 | 67 ± 14 | 94 ± 15 | 100 ± 23 |
| O35678 | Monoacylglycerol lipase | MGLL | 67 ± 8 | 39 ± 6 | 16 ± 6 |
| O08914 | Fatty-acid amide hydrolase 1 | FAAH | 64 ± 9 | 24 ± 4 | 21 ± 8 |
| Q6PE15 | α−β hydrolase 10 | ABHD10 | 63 ± 9 | 145 ± 25 | 49 ± 9 |
| Q91V12 | Cytosolic acyl coenzyme A thioester hydrolase | ACOT7 | 58 ± 9 | - | - |
| Q99LR1 | α−β hydrolase 12 | ABHD12 | 57 ± 3 | 130 ± 10 | 315 ± 38 |
| O88851 | Putative hydrolase RBBP9 | RBBP9 | 52 ± 16 | 85 ± 12 | 43 ± 8 |
| Q8BVQ5 | Protein phosphatase methylesterase 1 | PME1 | 46 ± 6 | 29 ± 4 | 30 ± 14 |
| Q9QYR9 | Acyl-coenzyme A thioesterase 2 | ACOT2 | 42 ± 6 | 530 ± 57 | 206 ± 34 |
| Q8R146 | Acylamino-acid-releasing enzyme | APEH | 41 ± 3 | 95 ± 18 | 142 ± 10 |
| Q8K1N1 | Calcium-independent phospholipase A2-γ | PNPLA8 | 39 ± 6 | 87 ± 13 | 25 ± 7 |
| Q8VEB4 | Group XV phospholipase A2 | PLA2G15 | 32 ± 5 | 85 ± 8 | 197 ± 15 |
| Q7M759 | α−β hydrolase FAM108B1 | ABHD17B | 31 ± 5 | 59 ± 2 | 31 ± 5 |
| Q80YA7 | Dipeptidyl peptidase 8 | DPP8 | 30 ± 10 | 24 ± 3 | 37 ± 12 |
| Q8VCV1 | α−β hydrolase FAM108C1 | ABHD17C | 29 ± 4 | 21 ± 1 | 12 ± 1 |
| Q9Z1Q2 | Protein BAT5 | ABHD16A | 28 ± 5 | 139 ± 28 | 23 ± 9 |
| P97823 | Acyl-protein thioesterase 1 | LYPLA1 | 28 ± 3 | 77 ± 3 | 58 ± 10 |
| Q8K4F5 | α−β hydrolase 11 | ABHD11 | 26 ± 3 | 72 ± 9 | 30 ± 8 |
| O55137 | Acyl-coenzyme A thioesterase 1 | ACOT1 | 19 ± 7 | 450 ± 58 | 256 ± 77 |
| Q9ET22 | Dipeptidyl peptidase 2 | DPP7 | 19 ± 3 | 55 ± 3 | 159 ± 39 |
| P11214 | Tissue-type plasminogen activator | PLAT | 19 ± 1 | 15 ± 1 | - |
| Q99JW1 | α−β hydrolase FAM108A | ABHD17A | 18 ± 3 | 20 ± 1 | 16 ± 2 |
| Q3TRM4 | Neuropathy target esterase | PNPLA6 | 18 ± 4 | 98 ± 8 | 36 ± 12 |
| Q8BVG4 | Dipeptidyl peptidase 9 | DPP9 | 17 ± 4 | 24 ± 7 | 49 ± 9 |
| Q9DB29 | Isoamyl acetate-hydrolyzing esterase 1 homolog | IAH1 | 17 ± 3 | 12 ± 2 | 24 ± 3 |
| P21836 | Acetylcholinesterase | ACHE | 16 ± 2 | - | - |
| Q9EP89 | Serine beta-lactamase-like protein LACTB | LACTB | 16 ± 2 | 16 ± 2 | 15 ± 4 |
| Q7TMR0 | Lysosomal Pro-X carboxypeptidase | PRCP | 16 ± 1 | 174 ± 21 | 208 ± 25 |
| P16675 | Lysosomal protective protein | CTSA | 14 ± 3 | 204 ± 39 | 229 ± 18 |
| Q9R118 | Serine protease HTRA1 | HTRA1 | 14 ± 4 | - | - |
| Q3UFF7 | Lysophospholipase-like protein 1 | LYPLAL1 | 14 ± 3 | 32 ± 14 | 13 ± 5 |
| Q64514 | Tripeptidyl-peptidase 2 | TPP2 | 13 ± 3 | - | 8 ± 5 |
| Q80YA3 | Phospholipase DDHD1 | DDHD1 | 12 ± 1 | 7 ± 1 | - |
| Q9JIY5 | Serine protease HTRA2 | HTRA2 | 12 ± 1 | 8 ± 1 | 7 ± 1 |
| Q5XJY4 | Presenilins-associated rhomboid-like protein | PARL | 11 ± 1 | 30 ± 3 | 21 ± 1 |
| P54310 | Hormone-sensitive lipase | LIPE | 10 ± 1 | - | 25 ± 5 |
| Q80UX8 | α−β hydrolase 13 | ABHD13 | 8 ± 1 | 19 ± 3 | 5 ± 1 |
| Q91WC9 | Sn1-specific diacylglycerol lipase β | DAGLB | 8 ± 1 | 13 ± 2 | 48 ± 8 |
| Q8VDG7 | Platelet-activating factor acetylhydrolase 2 | PAFAH2 | 7 ± 3 | 49 ± 3 | 26 ± 6 |
| O35448 | Lysosomal thioesterase PPT2 | PPT2 | 6 ± 2 | 66 ± 10 | 51 ± 13 |
| Q9CZN8 | Glutamyl-tRNA(Gln) amidotransferase subunit A homolog | QRSL1 | 6 ± 1 | 7 ± 1 | - |
| Q920A5 | Retinoid-inducible serine carboxypeptidase | SCPEP1 | 6 ± 1 | 35 ± 3 | 32 ± 5 |
| P97819 | 85 kDa calcium-independent phospholipase A2 | PLA2G6 | 5 ± 2 | - | - |
| Q60963 | Platelet-activating factor acetylhydrolase | PLA2G7 | 5 ± 1 | 41 ± 5 | 17 ± 1 |
| Q6WQJ1 | Sn1-specific diacylglycerol lipase α | DAGLA | 5 ± 1 | - | - |
| Q9QXM0 | α−β hydrolase 2 | ABHD2 | - | 49 ± 3 | 8 ± 3 |
| Q91ZH7 | α−β hydrolase 3 | ABHD3 | - | 64 ± 13 | 16 ± 9 |
| Q8VD66 | α−β hydrolase 4 | ABHD4 | - | 80 ± 8 | 35 ± 11 |
| Q9QYR7 | Acyl-coenzyme A thioesterase 3 | ACOT3 | - | 30 ± 12 | 16 ± 9 |
| Q8BWN8 | Acyl-coenzyme A thioesterase 4 | ACOT4 | - | 10 ± 3 | 15 ± 11 |
| Q6Q2Z6 | Acyl-coenzyme A thioesterase 5 | ACOT5 | - | 15 ± 6 | 30 ± 12 |
| Q32Q92 | Acyl-coenzyme A thioesterase 6 | ACOT6 | - | 19 ± 7 | 20 ± 15 |
| O35298 | Acyloxyacyl hydrolase | AOAH | - | - | 18 ± 3 |
| Q03311 | Cholinesterase | BCHE | - | 5 ± 1 | - |
| Q8R164 | Valacyclovir hydrolase | BPHL | - | 9 ± 1 | - |
| P28843 | Dipeptidyl peptidase 4 | DPP4 | - | - | 21 ± 13 |
| P16301 | Phosphatidylcholine-sterol acyltransferase | LCAT | - | 9 ± 1 | - |
| Q9Z0M5 | Lysosomal acid lipase/cholesteryl ester hydrolase | LIPA | - | 26 ± 2 | 24 ± 4 |
| P11152 | Lipoprotein lipase | LPL | - | - | 19 ± 7 |
| P47713 | Cytosolic phospholipase A2 | PLA2G4A | - | 8 ± 4 | 11 ± 1 |
| A2AJ88 | Patatin-like phospholipase domain-containing protein 7 | PNPLA7 | - | 54 ± 5 | 48 ± 8 |
| O88531 | Palmitoyl-protein thioesterase 1 | PPT1 | - | 8 ± 1 | - |
| Q3U213 | Protein SERAC1 | SERAC1 | - | 6 ± 1 | - |
| P70665 | Sialate O-acetylesterase | SIAE | - | 32 ± 6 | 34 ± 6 |
